# PEP-19 modulates calcium binding to calmodulin by electrostatic steering

**DOI:** 10.1038/ncomms13583

**Published:** 2016-11-23

**Authors:** Xu Wang, John A. Putkey

**Affiliations:** 1 Department of Biochemistry and Molecular Biology, McGovern Medical at UTHealth, 6431 Fannin, Houston, Texas 77030, USA

## Abstract

PEP-19 is a small protein that increases the rates of Ca^2+^ binding to the C-domain of calmodulin (CaM) by an unknown mechanism. Although an IQ motif promotes binding to CaM, an acidic sequence in PEP-19 is required to modulate Ca^2+^ binding and to sensitize HeLa cells to ATP-induced Ca^2+^ release. Here, we report the NMR solution structure of a complex between PEP-19 and the C-domain of apo CaM. The acidic sequence of PEP-19 associates between helices E and F of CaM via hydrophobic interactions. This allows the acidic side chains in PEP-19 to extend toward the solvent and form a negatively charged surface that resembles a catcher's mitt near Ca^2+^ binding loop III of CaM. The topology and gradients of negative electrostatic surface potential support a mechanism by which PEP-19 increases the rate of Ca^2+^ binding to the C-domain of CaM by ‘catching' and electrostatically steering Ca^2+^ to site III.

## Abstract (web summary)

The protein PEP-19 increases the rates of calcium binding to calmodulin. Here, the authors report the structure of PEP-19 bound to the C-terminal domain of calmodulin, and are able to propose a mechanism for the observed increased calcium association rate.

## Graphical abstract

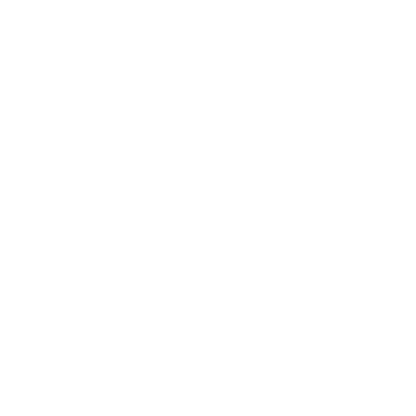

Calmodulin (CaM) is a ubiquitous Ca^2+^ sensor protein that is highly conserved and essential for the survival and function of all eukaryotic cells. CaM consists of globular N- and C-domains connected by a flexible linker that allows structural diversity in target protein recognition^1^,^2^,^3^. The C-domain of vertebrate CaM binds Ca^2+^ with slightly greater affinity (*K*_Ca50_≈2 μM) than the N-domain (*K*_Ca50_≈10 μM)^4^,^5^,^6^, but the most striking difference is that the rates of Ca^2+^ association and dissociation at the C-domain are at least 30- to 150-fold slower than for the N-domain^7^,^8^,^9^. Thus, Ca^2+^ binding to the C-domain is rate-limiting for CaM to attain the fully Ca^2+^-saturated state. Clearly, proteins that could potentially modulate or tune the Ca^2+^ binding properties of CaM would enhance its ability to sense diverse Ca^2+^ signals that vary greatly in amplitude and frequency. Our search for such regulators of CaM signalling led us to a small, intrinsically disordered protein called PEP-19.

PEP-19 has no known intrinsic activity other than binding selectively to the C-domain of CaM via an IQ motif (IQXXXR(GX)XXX). Although originally identified in the central nervous system^10^, PEP-19 mRNA is also found in human bladder, kidney, prostate, uterus, thyroid, cardiac and adrenal tissues. Consistent with its role as a regulator of CaM signalling or RCS protein, PEP-19 exerts broad effects on diverse cellular processes. For example, PEP-19 null mice show a dramatic reduction in long-term plasticity at synapses between granule cell parallel fibres and Purkinje cells^11^, as well as altered cardiac excitability^12^. Overexpression of PEP-19 in PC12 cells increases neurite outgrowth^13^, and premature neuronal differentiation is seen in transgenic mice with three copies of the PEP-19 gene^14^. In addition, PEP-19 has anti-apoptotic activity when expressed in PC12 and HEK293T cells^15^,^16^, and it provides protection against Ca^2+^ overload in cortical neurons^16^. Finally, PEP-19 sensitizes HeLa cells to ATP-dependent Ca^2+^ release^17^.

We discovered that PEP-19 has the remarkable ability to increase both the Ca^2+^ *k*_on_ and *k*_off_ rates at the C-domain of CaM up to 40-fold with little effect on Ca^2+^ binding affinity^9^, and that this property is dependent on an acidic sequence located adjacent to the IQ motif. Moreover, the acidic sequence is required for PEP-19 to sensitize HeLa cells to ATP-dependent Ca^2+^ release. Deleting or mutating the acidic sequence by, for example, converting Pro37 to Gly (PEP(P37G)) does not prevent PEP-19 from binding to CaM, but greatly decreases its ability to both modulate Ca^2+^ binding to C-domain of CaM and to sensitize HeLa cells to ATP-induced Ca^2+^ release^17^. This demonstrates that simply binding to CaM does not account for all the biological activities of PEP-19, and that tuning Ca^2+^ binding to CaM via the cooperative properties of its acidic sequence and IQ motif is an important mechanism by which PEP-19 impacts at least some CaM signalling pathways.

The ability of PEP-19 to increase the rate-limiting association of Ca^2+^ with the C-domain of apo CaM has the potential to regulate the temporal binding of CaM to a wide spectrum of Ca^2+^-dependent target proteins. This led us to determine the NMR solution structure of the PEP-19:apo C-CaM complex to reveal the molecular mechanism by which PEP-19 modulates the rate of Ca^2+^ binding to C-CaM. The structure, coupled with analysis the mutant PEP(P37G), demonstrate the importance of properly orienting the acidic residues in PEP-19 within the complex to increase the negative electrostatic surface potential (ESP), and electrostatically steer Ca^2+^ to site III in the C-domain of CaM.

## Results

### Use of C-CaM to study interactions between PEP-19 and CaM

We focused our structural analysis on interactions between PEP-19 and apo CaM since increasing the rate-limiting association of Ca^2+^ with the C-domain of CaM has the potential to increase its rate of association with other Ca^2+^-dependent target proteins. We used the isolated C-domain of CaM (C-CaM) since we showed previously that PEP-19 binds preferentially to the C-domain^6^,^18^, and that PEP-19 has the same effect on the Ca^2+^ binding properties of both intact CaM and C-CaM^19^. The extent of preferential binding of PEP-19 to the C-domain of apo CaM is emphasized in Supplementary Fig. 1a. PEP-19 has essentially no effect on backbone amide chemical shifts in the N-domain of apo CaM with an average change of only 0.007 p.p.m. for residues 3–73. In contrast, the average chemical shift perturbation across residues 81–148 in the C-domain of CaM is 0.27 p.p.m. Supplementary Fig. 1b,c show that the PEP-19 has the essentially same effects on the pattern and magnitude of backbone amide chemical shifts in both intact apo CaM and apo C-CaM, which suggests that the conformational change is also the same. Moreover, these changes are maximal at 1:1 molar ratio of PEP-19 to CaM or C-CaM. These data demonstrate that C-CaM is a valid structural model for the interaction between PEP-19 and CaM.

### Effects of apo C-CaM on the global structure of PEP-19

**Figure 1:**
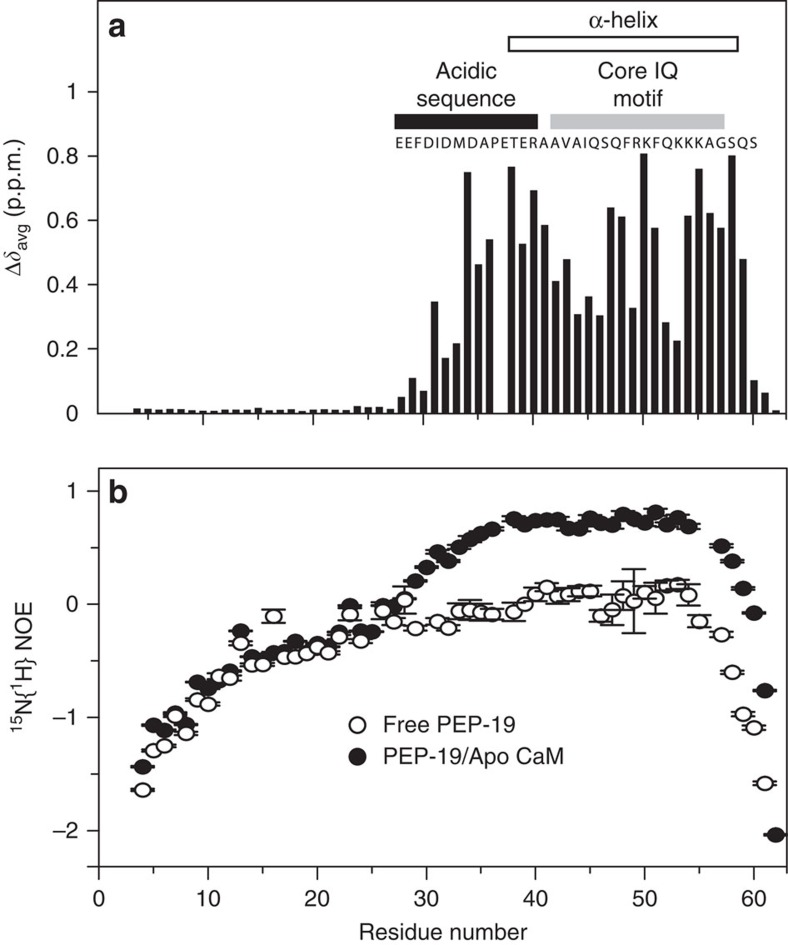
Structural transitions in PEP-19 on binding to apo C-CaM are restricted to its acidic sequence and the core IQ motif. (**a**) Backbone amide chemical shift changes calculated using equation (1) in ^15^N-labelled PEP-19 on binding to apo C-CaM. (**b**) Steady-state ^15^N{^1^H} NOEs of ^15^N free PEP-19 (open circles) and ^15^N PEP-19 bound to apo C-CaM (closed circles). The ^15^N{^1^H} NOE values and the uncertainty of NOE values (error bars) were determined as described in the ‘Methods' section.

Figure 1a shows that association with apo C-CaM has no effect on amide chemical shifts of residues 1–29 in ^15^N-labelled PEP-19, but large changes are observed for residues 30–58, which span both the acidic sequence and core IQ motif (see full spectra in Supplementary Fig. 2). The open bar in Fig. 1a indicates that Glu38 to Ala58 in PEP-19 are predicted to adopt α-helical secondary structure on binding to apo C-CaM based on the chemical shift index (CSI). Although binding to apo C-CaM causes large amide chemical shift changes for residues 30–36 in the acidic sequence of PEP-19, the CSI assigns this region to neither α-helix nor β-sheet secondary structure. Heteronuclear ^15^N{^1^H} nuclear Overhauser effects (NOEs) for free PEP-19 shown in Fig. 1b are predominantly negative, which is consistent with a highly flexible, intrinsically disordered protein. The ^15^N{^1^H} NOE values for residues Phe30–Ala36 in PEP-19 bound to apo C-CaM, show a gradual increase from 0 to 0.8, then the ^15^N{^1^H} NOE values remain high for residues Glu38–Ala58, which is consistent with a well-ordered α-helix.

### Structure determination of the PEP-19:apo C-CaM complex

**Figure 2:**
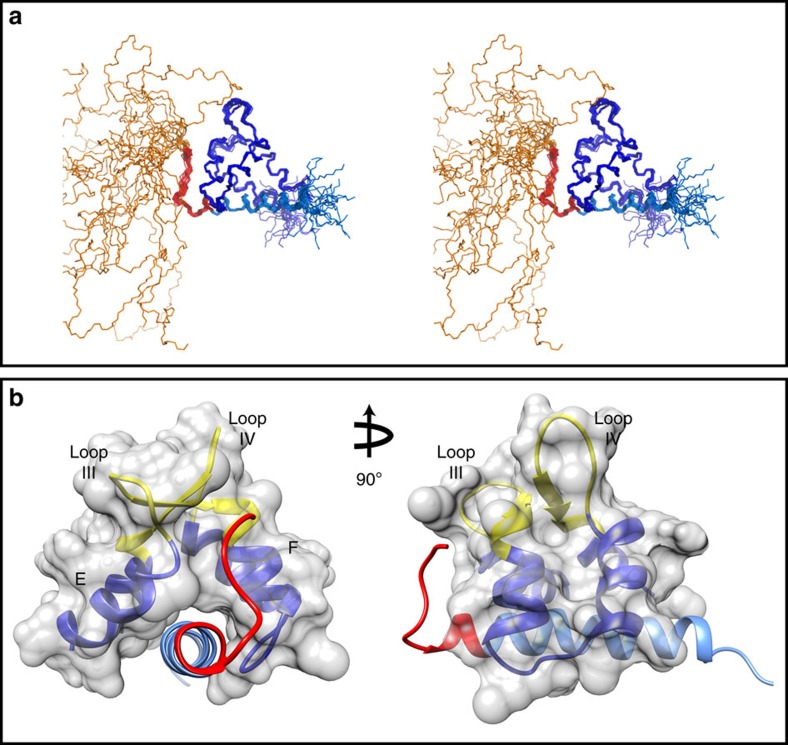
Three-dimensional solution structure of the PEP-19:apo C-CaM complex. (**a**) An stereo representation of an overlay of all backbone atoms for the final ensemble of 20 lowest energy conformers. Apo C-CaM is shown in dark blue. The disordered N terminus (aa 1–29), acidic sequence (aa 30–40) and IQ motif (aa 41–62) are shown in gold, red and light blue, respectively. (**b**) Ribbon representations for the structured regions of average minimized structure excluding the disordered N-terminal region of PEP-19. The colour scheme is the same as in **a**. Roman numerals III and IV indicate the Ca^2+^ binding loops of EF-hands III and IV, respectively, which are shown in yellow. All other figures also use the averaged minimized structure for the ensemble.

Over 99% of side chain protons were assigned for the PEP-19:apo C-CaM complex. Table 1 shows restraints used for structure calculations as well as structural statistics for an ensemble of the 20 lowest energy structures. The restraints included 1,926 NOE distance restraints, 98 predicted hydrogen bond distance restraints, 162 ϕ and Ψ angles from TALOS and 60 backbone H-N residual dipolar couplings (RDCs). Figure 2a shows a stereo view of the 20 lowest energy structures. Consistent with the data in Fig. 1a,b, residues 1–29 in PEP-19 remain disordered when bound to apo C-CaM, and show no detectable medium or long range intramolecular or intermolecular NOEs. Figure 2b shows the minimized average structure of the ensemble, excluding the disordered N-terminal region in PEP-19. Apo C-CaM is shown in dark blue, the acidic sequence is in red and the IQ motif of PEP-19 is in light blue.

### Structural features of apo C-CaM bound to PEP-19

On the basis of the CSI and TALOS, secondary structural elements in apo C-CaM bound to PEP-19 include helix E (aa 81–92), helix F (aa 102–111), helix G (aa 118–128), helix H (aa 138–146), β-strand III (aa 99–101) and β-strand IV (aa 135–137). Residues 93–104 and 129–140 form Ca^2+^ binding loops III and IV, respectively. The global backbone conformation of apo C-CaM bound to PEP-19 conforms to a semi-open state, with a shallow hydrophobic groove to accommodate the helical IQ motif of PEP-19. Pairwise structural alignments show that the closest structural relatives to apo C-CaM bound to PEP-19 are structures of semi-open CaM bound to other IQ motif peptides and proteins. Supplementary Fig. 3 shows an overlay of backbone structures for C-CaM bound to intact PEP-19 and those of C-CaM derived from the crystal structures of CaM bound to the IQ motif in the cytoplasmic domain of the voltage-gated sodium channel Na_V_1.5 (ref. 20) or the IQ region of myosin V^21^. The structures are shown without PEP-19 or the IQ peptides to emphasize spatial similarity of the 4 helices in C-CaM. Supplementary Table 1 compares helical angles for Ca^2+^ binding sites III and IV in Ca^2+^ and apo CaM versus the semi-open conformation of apo CaM bound to PEP-19 or the IQ peptide from the Na_V_1.5 channel^20^. Interestingly, the semi-open conformation in these complexes is largely due to a conformational change in site III since the helical angles of site III in apo CaM bound to either PEP-19 or the IQ peptide from Na_V_1.5 are essentially identical to free Ca^2+^ bound CaM.

Two experiments argue against the semi-open conformation of apo C-CaM as the primary molecular mechanism by which PEP-19 increases the rate of Ca^2+^ association. First, a peptide encompassing the IQ motif of PEP-19, but lacking the acidic sequence, does not increase the Ca^2+^ *k*_on_ (ref. 6). Second, Supplementary Fig. 4 shows that the IQ peptide from the voltage-gated sodium channel Na_V_1.5 (aa 1,901–1,927), which lacks an acidic sequence but clearly induces a semi-open conformation in the C-domain of CaM^22^, has little effect on the rate of Ca^2+^ association.

Although the helical angle for site III of apo CaM bound to PEP-19 is more similar to Ca^2+^ CaM, the backbone conformation and side chain orientations for residues 93–104 in the Ca^2+^ binding loop III are much more similar to free apo CaM. The conformation of Ca^2+^ binding loop III in the PEP-19:apo C-CaM complex is fairly well defined with 85 medium and long range NOEs, and appears to be stabilized by a network of hydrogen bonds involving the side chain oxygens of Asp93 and backbone amides of residues 94–99 (also see the ‘Discussion' section). In contrast, only 18 medium and long range NOEs are observed in loop IV. This is consistent with amide line broadening observed for residues 129–134 due to chemical exchange, which was also seen for apo CaM bound to the IQ motif peptide of Na_V_1.5 (ref. 20).

### Interaction between C-CaM and the helical portion of PEP-19

**Figure 3:**
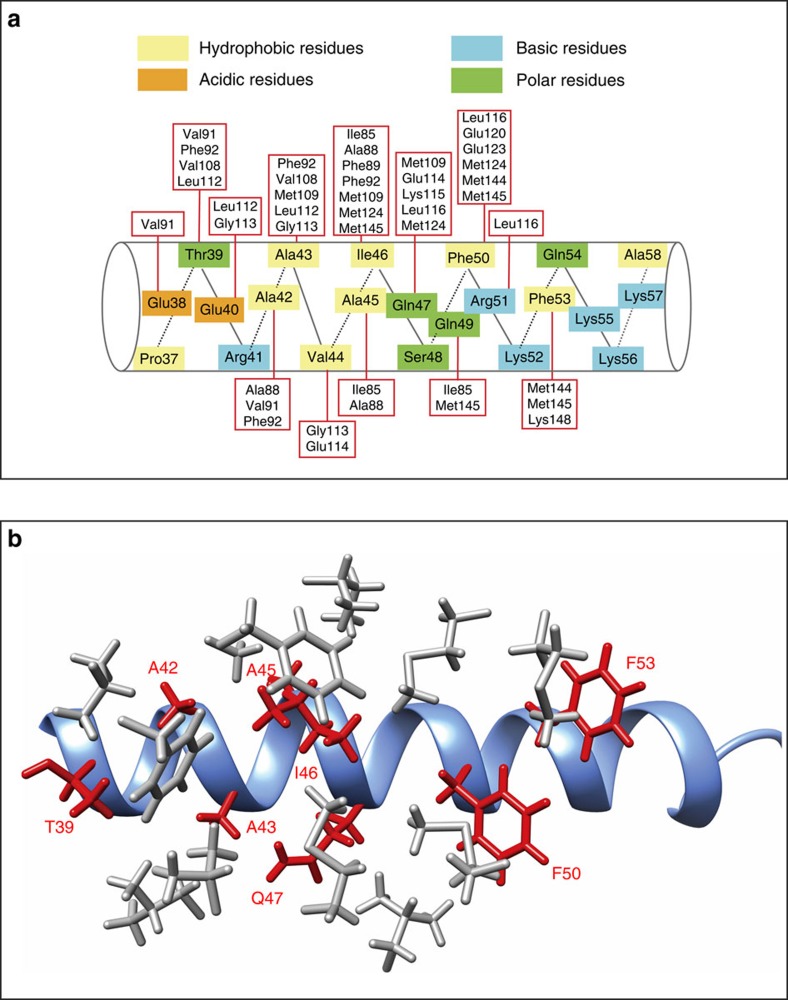
Interactions between the helical portion of PEP-19 and apo C-CaM. (**a**) Intermolecular NOEs observed between residues in the helical segment of PEP-19 and residues in apo C-CaM. (**b**) A ribbon diagram of the helical portion of PEP-19 (light blue) with side chains (red) that form hydrophobic interactions with residues in apo C-CaM (grey).

PEP-19 binds to C-CaM in a parallel orientation with the N-terminal residues interacting with helices E and F of Ca^2+^ binding site III, while the C-terminal residues interact with helices G and H in Ca^2+^ binding site IV. The average total buried surface area is 2,157 Å^2^ for the five lowest energy conformers in the ensemble. Figure 3a summarizes residues in the helical portion of PEP-19 that form intermolecular NOEs with residues in C-CaM. The majority of NOEs arise from hydrophobic interactions involving non-polar atoms in Thr39, Ala42, Ala43, Ala45, Ile46, Gln47, Phe50 and Phe53 in PEP-19. Figure 3b shows that these residues (red) are predominantly located on one surface of the helical segment in PEP-19. They are deeply buried in the complex, with 89% of their side chain surfaces excluded from solvent, which allows them to form hydrophobic interactions primarily with Phe89, Phe92, Val108, Met109, Met124, Met144 and Met145 in C-CaM, which line the shallow hydrophobic groove (see grey side chains in Fig. 3b). In addition to these hydrophobic interactions, electrostatic interactions include hydrogen bonds between Gln47 in the IQ dipeptide of PEP-19 and backbone atoms of Ile112 and Glu114 in the linker between helices F and G in apo C-CaM, as well as ionic bonds between the side chains of Arg51 in PEP-19 and Glu114 in C-CaM.

The importance of these residues for binding to CaM is supported by a study using synthetic peptides that span residues 36–60 in the IQ motif of PEP-19 (ref. 23). Peptides with Ile46 and Gln47 changed to Gly, as well as the single mutations A42D, F50G, R51K, K56A and K57A all inhibited their ability to inhibit CaM activation of nNOS at low Ca^2+^. Similarly, introducing the mutations Q47N and S48T effectively prevented intact PEP-19 from binding to CaM, and from conferring resistance to Ca^2+^-mediated cytotoxicity^16^.

### Interactions between C-CaM and the acidic loop in PEP-19

**Figure 4:**
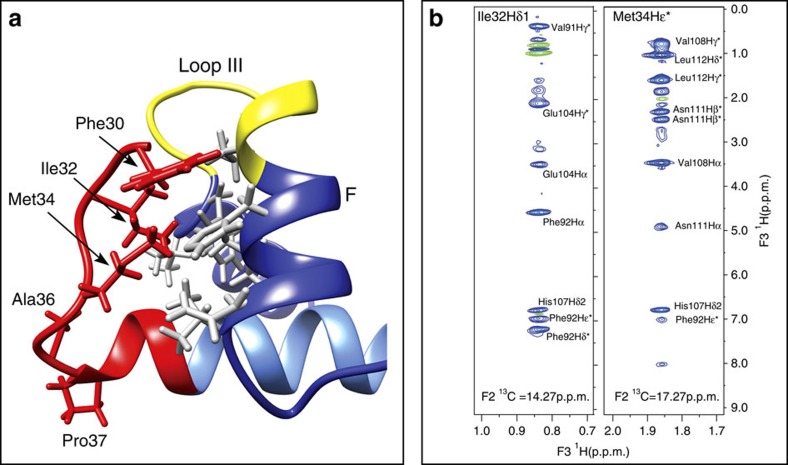
Interactions between the acidic sequence of PEP-19 and helices E and F of Ca^2+^ binding site III in apo C-CaM. The acidic sequence is shown in red, helices E and F in C-CaM are shown in dark blue, and Ca^2+^ binding loop III is in yellow. (**a**) Phe30, Ile32 and Met34 in the extended coil region in PEP-19 form hydrophobic interactions with residues in C-CaM (grey) that are at the interface between helices E and F in Ca^2+^ binding site III. (**b**) Selected planes acquired from F_1_-filtered, F_3_-edited NOESY-HSQC using ^13^C, ^15^N PEP-19 bound to unlabelled C-CaM. The planes highlight numerous intermolecular NOEs from Hδ1 of Ile32 and Hɛ of Met34 in PEP-19 to residues in C-CaM.

Figures 2b and 4a show that the acidic sequence in PEP-19 (aa 30–40 in red) associates with Ca^2+^ binding site III. Residues 30–36 in PEP-19 form an extended coil structure that runs between helices E and F of Ca^2+^ binding site III. The coil segment terminates at Pro37, and residues 38–40 of the acidic sequence form the N terminus of the α-helix in PEP-19. The buried surface between the acidic sequence and C-CaM is 725 Å^2^, which is 30% of the total buried surface area in the complex.

Figure 4a shows that the side chains of Phe30, Ile32 and Met34 of PEP-19 form a hydrophobic plug that inserts between helices E and F of Ca^2+^ binding site III to stabilize the extended coil segment of PEP-19. The side chains of these three residues account for 89% of the buried surface area in the acidic sequence of PEP-19. A total of 39 intermolecular NOEs are observed between C-CaM and the side chains of Phe30, Ile32 and Met34 in PEP-19. This extensive network of NOEs is illustrated in Fig. 4b, which shows selected planes acquired from F_1_-filtered, F_3_-edited NOESY-HSQC using ^13^C, ^15^N PEP-19 bound to unlabelled C-CaM. The Hδ1 of Ile32 in PEP-19 shows intermolecular NOEs to Val91, Phe92, Glu104 and His107 in C-CaM. The Hɛ of Met34 in PEP-19 shows intermolecular NOEs to Phe92, His107, Val108, Asn111 and Leu112 in C-CaM (see grey side chains in Fig. 4a). Most of these residues in C-CaM also form intermolecular NOEs with residues in the helical portion of PEP-19, especially Ile46 of the IQ dipeptide. This network of hydrophobic interactions may provide allosteric communication between the acidic loop and helical region of PEP-19 bound to apo C-CaM.

### PEP-19 generates negative ESP near Ca^2+^ binding loop III

**Figure 5:**
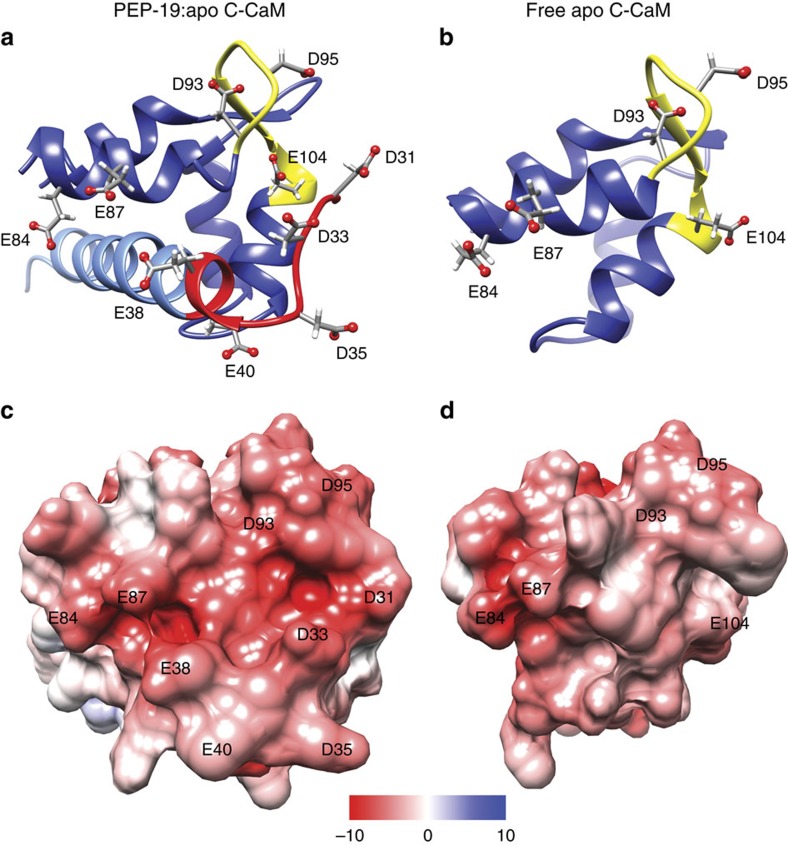
The acidic sequence of PEP-19 greatly increases the negative ESP near Ca^2+^ binding site III of apo C-CaM. (**a**,**b**) Ribbon diagrams for the PEP-19:apo C-CaM complex and free C-CaM, respectively. Dark blue is C-CaM, yellow is Ca^2+^ binding loop III, red and light blue are the acidic sequence and core IQ motif in PEP-19, respectively. (**c**,**d**) Solvent excluded surfaces that are coloured based on ESP.

Figure 5a,b focus on Ca^2+^ binding site III in free apo C-CaM and the PEP-19:apo C-CaM complex. As mentioned above, the conformation of loop III is similar in both structures. While the angle formed by helices E and F is quite different between the structures, this alone does not account for the altered Ca^2+^ binding kinetics induced by PEP-19. This led us to evaluate the effect of PEP-19 on ESP. Figure 5a shows that the side chains of Asp35 and Glu40 in the acidic sequence of PEP-19 extend away from Ca^2+^ binding loop III. However, the side chains of Asp31, Asp33 and Glu38 lie along the surface of the acidic sequence that opposes helix E and the first three residues in loop III of C-CaM (aa 93–95). Figure 5c shows that the acidic residues in PEP-19 combine with Glu84, Glu87, Asp93 and Asp95 in C-CaM to form a large surface with significant negative ESP. This contrasts with the more neutral ESP shown for free apo CaM in Fig. 5d. A view of loop III from another angle in Supplementary Fig. 5 shows a foci of negative ESP is also formed from the side chains of Asp93 and Glu104.

### Effects of PEP(P37G) mutation on the acidic sequence

We showed previously that replacing Pro37 with Gly (PEP(P37G)) greatly inhibits the ability of PEP-19 modulate Ca^2+^ binding to CaM, and to sensitize HeLa cells to ATP-dependent Ca^2+^ release^17^. Figure 4a shows that Pro37 terminates the helical segment of PEP-19, and intramolecular NOEs show that Pro37 and Ala36 interact with neighbouring residues to stabilize the transition between helical and coiled segments in PEP-19. Thus, a plausible hypothesis is that conversion of Pro37 to Gly confers a high degree of flexibility that inhibits residues 30–36 from properly interacting with CaM. To test this hypothesis, we used chemical shift perturbation mapping and paramagnetic relaxation enhancement (PRE) to determine the effects of mutation of Pro37 on the structure and position of the acidic sequence.

Supplementary Fig. 6a shows that conversion of Pro37 to Gly causes relatively small amide chemical shift perturbations in free PEP-19 that are restricted to residues near Pro37. In contrast, Supplementary Fig. 6b shows that when bound to apo C-CaM, large chemical shift differences between PEP-19 and PEP(P37G) extend the length of the acidic sequence from Asp31 to Glu40. In addition, Supplementary Fig. 7a shows significantly reduced amide ^1^H chemical shift dispersion for Asp31 to Glu40 in PEP(P37G) versus PEP-19 when bound to C-CaM, which suggests that the mutation destabilizes the acidic sequence. This is consistent with Supplementary Fig. 7b, which shows that residues 30–40 in PEP(P37G) bound to apo C-CaM have the greatest decrease in ^15^N{^1^H} NOEs relative to the native complex.

**Figure 6:**
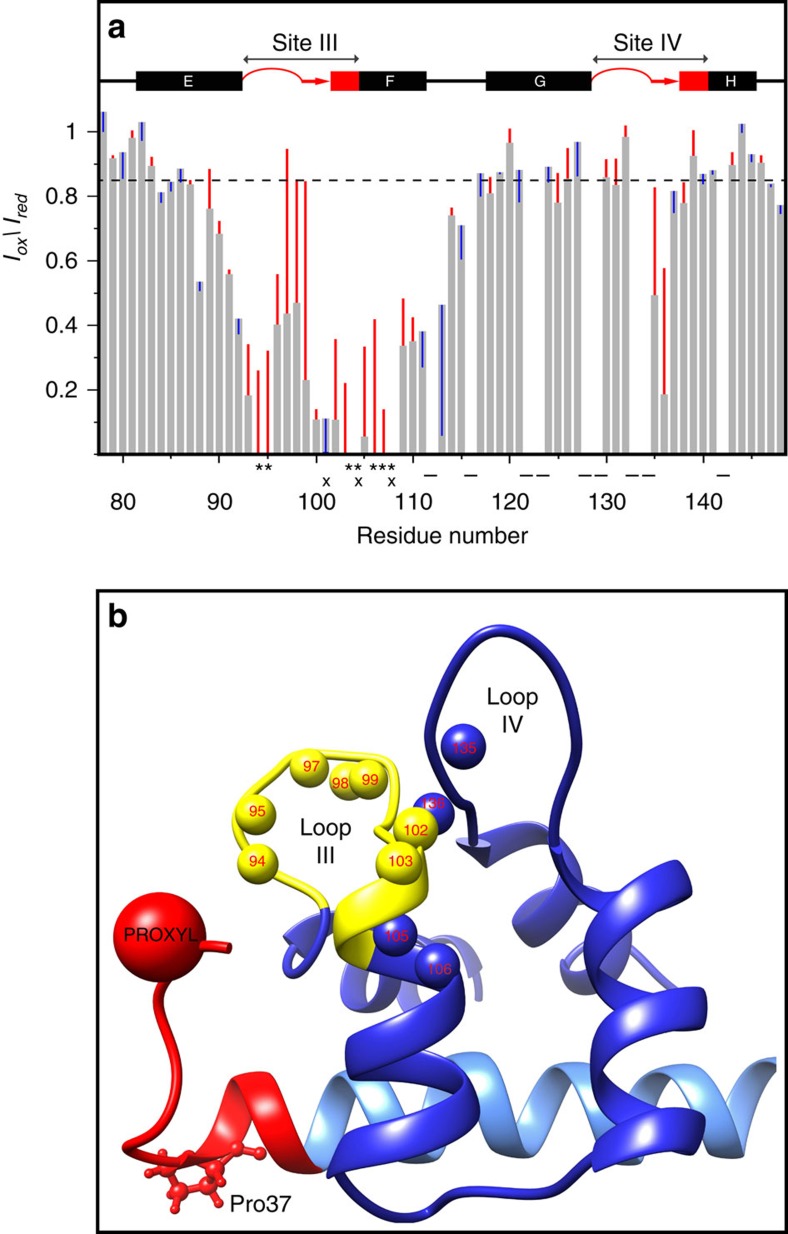
Mutation of Pro37 to Gly in PEP-19 increases the distance between the acidic sequence and Ca^2+^ binding site III in apo C-CaM. Asp31 in PEP-19 and PEP(P37G) was mutated to Cys and spin labelled for PRE analysis as described in the methods. The grey bars in (**a**) the normalized amide *I*_ox_/*I*_red_ intensity ratios derived from ^1^H, ^15^N HSQC spectrum of apo C-CaM when bound to PEP-19(SL). The red and blue vertical lines indicate an increase or decrease, respectively, in *I*_ox_/*I*_red_ due to mutation of Pro37 to Gly. The *I*_ox_/*I*_red_ could not be calculated for residues indicated by (−) due to spectral overlap. Amide cross peaks for residue indicated by (*) are line broadened beyond detection when C-CaM is bound to PEP-19(SL), while those indicated by (x) are line broadened beyond detection when bound to PEP(P37G)SL. (**b**) The location of amides (small balls) for residues in apo C-CaM that show the greatest increase in distance from the PROXYL probe with an increase in *I*_ox_/*I*_red_ of greater than 0.2 due to mutation of Pro37 to Gly. Calcium binding loop III is shown in yellow.

We next used PRE to determine if mutation of Pro37 to Gly in PEP-19 increases the distance between the acidic sequence and apo C-CaM. As described in the ‘Methods' section, Asp31 in both PEP-19 and PEP(P37G) was converted to Cys as a site for covalent modification with PROXYL to yield spin-labelled PEP-19SL and PEP(P37G)SL, respectively. The PRE effect was measured as the normalized cross-peak intensity ratio (*I*_ox_/*I*_red_) of each amide in the oxidized and reduced states. The grey bars in Fig. 6a show the PRE effect on backbone amides in ^15^N-labelled apo C-CaM bound to PEP-19SL. Similar to Battiste and Wagner^24^, a PRE effect was indicated by *I*_ox_/*I*_red_ ratios of <0.85 (see dashed horizontal line). The pattern of amide *I*_ox_/*I*_red_ ratios correlates well with the location of the spin label on residue 31 near Ca^2+^ binding loop III. Residues with amide *I*_ox_/*I*_red_ ratios <0.2, or that are broadened beyond detection by the oxidized spin label, include Asp93, Lys94 and Asp95, which are the first three residues in Ca^2+^ binding loop III, and Ile100 through Val108, which form β-strand III and extend into helix F. The *I*_ox_/*I*_red_ ratios of <0.5 are seen for Gly96 to Tyr99 in loop III. A significant PRE effect is also seen for Gln135 and Val136, which is consistent with the location of these two residues in a short beta strand in site IV that forms a beta sheet with residues in site III.

The coloured vertical lines in Fig. 6a show differences in *I*_ox_/*I*_red_ for ^15^N-labelled apo C-CaM when bound to PEP(P37G)SL versus PEP-19SL. A red line indicates that the distance between the corresponding backbone amide in C-CaM and the spin label bound to Cys31 is increased by mutation of Pro37 to Gly. Conversely, a blue line indicates a decreased distance. Large increases in *I*_ox_/*I*_red_ ratios are seen for residues in Ca^2+^ binding loop III, helix F and β-strand IV when apo C-CaM is bound to PEP(P37G)SL versus PEP-19SL. Figure 6b shows the position of these amide protons in the solution structure of the PEP-19:apo C-CaM complex. Taken together, these data demonstrate that conversion of Pro37 to Gly destabilizes the interaction between the acidic sequence and apo C-CaM, to increase the average distance between the acidic sequence and Ca^2+^ binding site III, thus altering the orientation of the acidic side chains in PEP-19 relative to C-CaM, and likely affecting the negative ESP near Ca^2+^ binding site III.

## Discussion

We report here the first structure of a complex between apo CaM and a protein that regulates the activity of CaM, instead of a target protein that is regulated by CaM. Our goal was to identify molecular mechanisms by which PEP-19 modulates Ca^2+^ binding to CaM. We focused on the apo state since increasing the rate of Ca^2+^ binding to apo CaM by PEP-19 has the potential to regulate the temporal binding of CaM to a wide spectrum of Ca^2+^-dependent target proteins. Structural features of the acidic sequence of PEP-19 were of special interests since it is necessary to both modulate Ca^2+^ binding to CaM and to sensitize HeLa cells to ATP-dependent Ca^2+^ release^17^.

First, this is fuzzy complex^25^ since residues 1–29 in PEP-19 remain disordered when bound to apo C-CaM. Although this disordered sequence is not required to modulate Ca^2+^ binding to CaM^6^, it may interact with other cellular proteins since residues 10–20 have a high propensity to form a β-turn based on analysis with ProtScale ( http://web.expasy.org/protscale/). Second, the IQ motif (residues 38–58) adopts a regular α-helix that lies along the shallow hydrophobic groove in semi-open apo C-CaM. Finally, and most significantly, residues 30–37 of the acidic sequence in PEP-19 adopt an extended coil that associates with helices E and F in Ca^2+^ binding site III of CaM primarily via a hydrophobic plug formed from Phe30, Ile32 and Met34 in PEP-19, which allows the acidic residues to extend toward the solvent.

To gain mechanistic insights from the structure, it is important to discuss the characteristics of cooperative Ca^2+^ binding to sites III and IV in CaM. Structural dynamics at low Ca^2+^ levels support the idea that the first Ca^2+^ binds preferentially to site IV, but with rapid *k*_on_ and *k*_off_ rates^26^. Since the overall rate of association of two Ca^2+^ ions to the C-domain is slow, binding the second Ca^2+^ to site III is rate-limiting^6^ but locks both Ca^2+^ in place. The slow kinetics of Ca^2+^ binding to site III may be due to an intra loop network of hydrogen bonds that surround the carboxyl group of Asp93 at position 1 in Ca^2+^ binding loop III, which must rearrange to allow the side chain of Asp93 to coordinate Ca^2+^. Association of the acidic sequence with Ca^2+^ binding site III places it in logical position to enhance the rate-limiting association of Ca^2+^ with the C-domain. However, there are no structural features of Ca^2+^ binding loop III in the PEP-19:apo C-CaM complex that would clearly account for the increased rate of Ca^2+^ association. In particular, the intra loop hydrogen bonds persist in the PEP-19:apo C-CaM complex, since side chain oxygens of Asp93 are within 2–3 Å from backbone amides of residues 94–99. Instead, we hypothesize that the primary mechanism for increasing the rate of Ca^2+^ association involves increasing the negative ESP to steer Ca^2+^ to site III.

**Figure 7:**
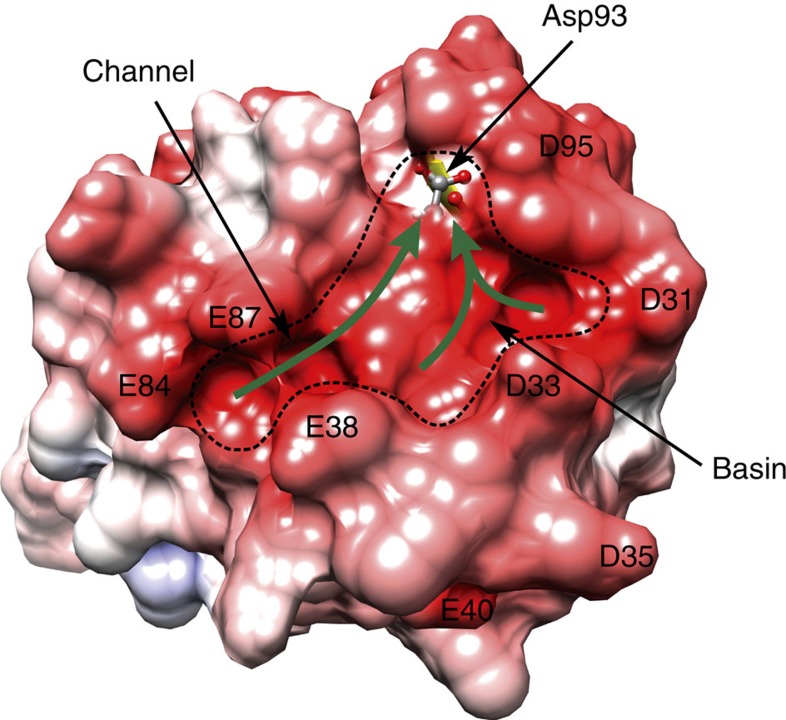
Steering of Ca^2+^ to Asp93 in Ca^2+^ binding loop III. The topological features (channel and basin) near Ca^2+^ binding site III in the PEP-19:apo C-CaM complex are outlined by the dashed line. The surface contributed by Asp93 in C-CaM is shown as transparent, and is located in negatively charged basin. The green arrows illustrate funneling of Ca^2+^ to Asp93 guided by electrostatic steering and surface topology.

As described in the results section, acidic residues derived from C-CaM and PEP-19 generate a large surface with significant negative ESP. Figure 7 shows that the topology of this surface includes a channel formed between the side chains of Glu84 and Glu87 in C-CaM and Glu38 in PEP-19. The channel leads to a basin that resembles a catcher's mitt that is surrounded by Asp31, Asp33 and Glu38 from PEP-19, and Asp93 and Asp95 from C-CaM. This region of negative ESP could stabilize an initial encounter complex between Ca^2+^ and the PEP-19:apo C-CaM complex and allow transient two-dimensional diffusion of Ca^2+^ that is guided by the channel and basin surface topology and electrostatic gradients. An additional important feature is the location of Asp93, which is the first ligand for Ca^2+^ in loop III. Figure 7 shows that the backbone oxygen and beta protons of Asp93 contribute to formation of the negatively charged basin. As illustrated by the green arrows in Fig. 7, we propose that electrostatic gradients and surface topology funnel or steer Ca^2+^ to Asp93, and that this helps overcome the energy barrier presented by the intra loop hydrogen bonding network involving Asp93, thereby increasing the rate of Ca^2+^ binding and transition to the Ca^2+^ bound form. Similar electrostatic steering to Asp93 and Glu104 may be supported by the negative ESP shown in Supplementary Fig. 5.

It is well established that electrostatic interactions can increase the rate of protein-protein and protein-ligand association by orders of magnitude^27^,^28^. A specific role for ESP in modulating the kinetics of Ca^2+^ binding to CaM by PEP-19 is supported by several observations, including our mutation analysis^17^. Mutation of Asp31 or Asp33 to Ala, or Pro37 to Gly all significantly inhibit the ability of PEP-19 to modulate Ca^2+^ binding, but mutation of Asp35 has little effect. This is consistent with Fig. 7, which shows that Asp31 and Asp33 surround the negatively charged basin, while Aps35 extends away from site III. Mutation of Pro37 to Gly has the greatest effect on the ability of PEP-19 because it destabilizes the interaction between the acidic sequence and site III of CaM, thereby affecting the relative position of multiple acidic residues. A causal relationship between negative ESP and fast Ca^2+^ *k*_on_ rates is also supported by EF-hands I, II and IV, which all have fast rates of Ca^2+^ association^7^,^8^,^9^, and all have extensive negatively charged ESP near the respective loops as shown in Supplementary Fig. 6. Finally, Martin *et al*.^29^ showed that acidic residues greatly affect the rate of Ca^2+^ association with EF-hand motifs even though they do not directly participate in Ca^2+^ binding. In this case, neutralization of acidic residues in the first EF-hand Ca^2+^ binding site of calbindin 9K decreased the Ca^2+^ *k*_on_ by 45-fold. We propose that PEP-19 achieves the inverse by adding negative charge to increase the rate of Ca^2+^ association.

**Figure 8:**
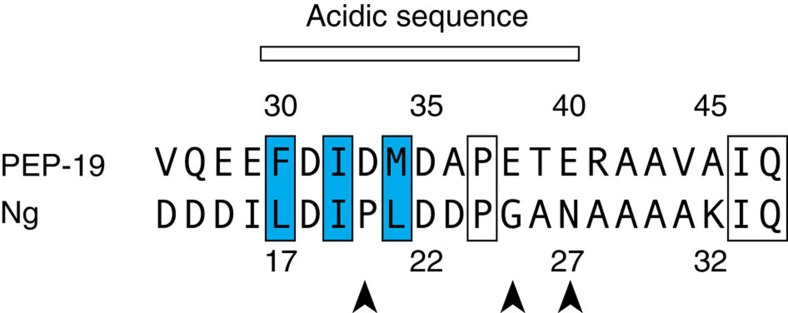
Comparison of acidic sequences in PEP-19 and Ng. Blue boxes indicate residues that anchor the acidic sequence of PEP-19 to Ca^2+^ binding site III. Arrowheads indicate the position of acidic residues in the acidic sequences of PEP-19 and Ng.

It is well known that the Ca^2+^ binding affinity of CaM can be drastically increased on binding to Ca^2+^-dependent target peptides and proteins due to stabilizing the Ca^2+^-bound form and decreasing the rate of Ca^2+^ dissociation^7^,^30^,^31^. The data presented here provide an additional molecular mechanism to modulate Ca^2+^ binding to CaM that may be shared by other proteins. Neurogranin (Ng), for example, is a small IQ motif protein that also binds to CaM in the presence or absence of Ca^2+^, and has an acidic sequence near its IQ motif. We showed that Ng increases the rate of Ca^2+^ dissociation from the C-domain of CaM to a similar extent as PEP-19, but has a lesser effect on the rate of Ca^2+^ association^32^. Thus, Ng decreases the affinity of Ca^2+^ binding to the C-domain of CaM. Figure 8 compares acidic sequences that extend N-terminal from the IQ dipeptide of PEP-19 and Ng. Positions corresponding to Phe30, Ile32 and Met34 in PEP-19, which anchor the acidic sequence to Ca^2+^ binding site III in CaM, are also hydrophobic residues in Ng. On the basis of this, we predict that the global structure of the Ng:apo C-CaM complex will be very similar to the PEP-19:apo C-CaM complex. However, the distribution of acidic residues in Ng and PEP-19 are different, which we predict confers different Ca^2+^ tuning properties. Another potential use of the acid/IQ motif is not as free regulator of CaM signalling, but as a module within the context of a larger Ca^2+^-dependent CaM target protein. In this scenario, the acidic/IQ motif would provide a docking site for apo CaM to modulate overall Ca^2+^-sensitivity, and allow CaM to reversibly transition to an adjacent Ca^2+^-dependent site to regulate protein activation/inactivation in response to specific Ca^2+^ signals.

In summary, the structure reported here shows a novel mode of interaction between apo CaM and a regulator of CaM signalling, and provides a molecular mechanism for modulation of Ca^2+^ binding to CaM that may be shared by other proteins. From a broader perspective, the high content of polar and charged residues in intrinsically disordered proteins makes them well suited to modulate ligand binding by changing the surface topology and electrostatic potential on binding to target proteins.

## Methods

### Expression and purification

Recombinant C-CaM and PEP-19 were expressed in bacteria and purified as described previously^9^. QuickChange II XL Site-directed Mutagenesis Kit (Agilent Technologies) was used to generate PEP-19 mutant proteins.

cDNAs encoding mammalian C-CaM or human PEP-19 were synthesized with optimal bacterial codon usage (DNA2.0), and sub cloned into a pET23d expression vector. Isotopically labeled C-CaM or PEP-19 were prepared by expression in *E. coli* BL21(DE3) or BL21(DE3)pLysS using M9 minimal media supplemented with 1 g l^−1^ ^15^NH_4_Cl as sole nitrogen and 2 g l^−1^ of unlabeled glucose or uniformly labeled ^13^C-glucose as carbon source.

After induction of C-CaM expression with IPTG, bacterial cells were collected by centrifugation, suspended in lysis buffer (25 mM Tris, 1 mM EDTA, pH 7.5), and then frozen. Frozen cell suspensions were thawed, sonicated and then centrifuged to yield a soluble fraction. Soluble protein was loaded on as semi preparative DEAE 5PW column (Waters) and eluted with a linear 0–300 mM KCl gradient. Ammonium sulfate ((NH_4_)_2_SO_4_) was slowly added to pooled fractions from the DEAE column to a final concentration of 2 M. After centrifugation to remove precipitated protein, the soluble protein fraction was loaded onto a phenyl sepharose column (GE Healthcare) and washed with loading buffer (25 mM Tris, 2 mM (NH_4_)_2_SO_4_, 1 mM EDTA, pH 7.5). Fractions containing C-CaM were eluted with a linear 2–0 mM (NH_4_)_2_SO_4_ gradient, then pooled, dialyzed against the 20 mM (NH_4_)HCO_3_, pH 7.5 for 24 h and lyophilized.

PEP-19 was expressed, and a soluble protein fraction prepared as described for C-CaM above, except that the lysis buffer was 20 mM MES, pH 6.5. The soluble protein fraction was passed through a Macro Q anion exchange column (Bio-Rad), which binds the majority of proteins, but not PEP-19. The pH of the fall-through from this first column was adjusted to pH 6 and loaded on a Hi-Trap SP cation exchange column (GE Healthcare), and eluted with a linear 0–500 mM KCl gradient. Fractions containing PEP-19 were loaded on a Vydac C-4 column and eluted with a 0–60% acetonitrile, 0.1% TFA. Fractions containing PEP-19 were pooled and lyophilized.

Protein concentrations were estimated using an extinction coefficient of ɛ 276 nm=0.18 ml^−1^mg^−1^ for C-CaM and ɛ 215 nm=0.59 ml^−1^mg^−1^ for PEP-19, or by using the BCA protein assay (Pierce).

### NMR methodology

The NMR samples for structure determination contained either 0.8 mM ^13^C, ^15^N C-CaM with excess unlabelled PEP-19 or ^13^C, ^15^N PEP-19 with excess unlabelled C-CaM. All NMR samples contained in 10 mM imidazole-d4 (pH 6.3), 100 mM KCl, 5 mM EDTA-d16 and 10 μM 2,2-dimethyl-2-silapentane-5-sulfonate (DSS), 95%H_2_O/5%D_2_O or 99.99% D_2_O. The sample used for RDC measurements also contained 5.0% C12E5 poly-ethylene-glycol/hexanol mixture. NMR data were acquired on a Bruker 600 MHz spectrometer equipped with a 5-mm TXI cyroprobe at 298 K.

Backbone assignments for bound PEP-19 were made using ^15^N HSQC, HNCA, HN(CO)CA, HNCACB, CBCA(CO)NH, HNCO and ^15^N-edited NOESY-HSQC experiments. Backbone assignments for bound apo C-CaM were obtained from published data^17^. Amides of Asp131 and Gly134 in apo C-CaM could not be observed due to conformational exchange on the μs to ms timescale on PEP-19 binding^17^. Aliphatic side chain assignments were made using 2D ^1^H-^13^C HSQC, 3D HN(CO)HBHA, (H)CC(CO)NH, H(CCCO)NH, CCH-TOCSY, HCCH-TOCSY and HCCH-COSY. ^1^H aromatic side chain assignments were obtained using 2D (HB)CB(CGCD)HD and (HB)CB(CGCE)HE. Intramolecular NOEs were assigned by 3D ^15^N NOESY-HSQC and ^13^C NOESY-HSQC. To reduce ambiguous intermolecular NOEs, 3D F_1_-^13^C/^15^N-filtered, F_3_-^13^C-edited NOESY spectra were acquired by using ^13^C, ^15^N C-CaM bound to unlabelled PEP-19 as well as ^13^C, ^15^N PEP-19 bound to unlabelled C-CaM. The mixing time for all NOESY experiments was 100 ms. H-N RDC measurements were performed using in-phase/anti-phase ^1^H, ^15^N HSQC experiment^33^.

Heteronuclear ^15^N{^1^H} NOEs were collected at 298 K at a ^15^N frequency of 60 MHz. The NMR samples included 0.5 mM ^15^N-labelled PEP-19, or PEP(P37G) with 1 mM unlabelled apo C-CaM. NOE values were measured from spectra with and without proton saturation recorded in an interleaved manner. Proton saturation was acquired using a 120° ^1^H pulse applied every 5 s. In the case of the NONOE spectra, a net relaxation delay of 5 s was employed, while a relaxation delay of 2 s before a 3 s proton presaturation period was employed for the NOE spectra. Heteronuclear ^15^N{^1^H} NOE values were determined by measuring the ratio of peak intensities in spectra acquired with and without proton saturation. The uncertainty for NOE values was evaluated using the standard deviation of the noise in empty spectral regions of the spectra^34^.

All NMR spectra were processed and analyzed using Topspin2.0 (Bruker) and FELIX 2004 (MSI, San Diego, CA, USA). ^1^H chemical shifts were referenced to DSS, and ^15^N and ^13^C chemical shifts were referenced indirectly using their respective gyromagnetic ratios^35^. The average amide chemical shift change was calculated using the following equation:


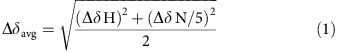


*Structure calculations*. Initial structures of the complex were generated using a torsion-angle molecular dynamics protocol with CNS 1.3 (refs 36, 37) using NOE distance restraints, hydrogen bond distance restraints predicted based on the secondary structure derived from the CSI^38^, and dihedral restraints predicted by TALOS^39^. Further structure refinement with the addition of backbone H-N RDC restraints was performed by XPLOR-NIH (version 2.42)^40^. Initial estimation for the axial component of the molecular alignment tensor (Da) and rhombicity (*R*) were obtained from the lowest energy structure calculated by CNS using PALES^41^. A family of 300 structures was generated, and the final ensemble of 20 structures with lowest energies was selected for analysis. Structural analysis and statistics were obtained using MOLMOL^42^ and PROCHECK-NMR^43^. PROCHECK-NMR shows 90.1% of residues in the most favored and 6.9% in additional allowed regions for the residues Ser81–Thr146 in C-CaM and Phe30–Ala58 in PEP-19, with 1.7 and 1.2% of residues in the generously allowed and disallowed regions, respectively.

Graphics and analyses were performed with PyMOL (DeLano, W.L (2002) the PyMOL Molecular Graphics System, DeLano Scientific, Palo Alto, CA, USA) and the UCSF Chimera package (Chimera is developed by the Resource for Biocomputing, Visualization and Informatics at the University of California, San Francisco (supported by NIGMS P41-GM103311). Accessible surface area was calculated with NACCESS (Hubbard, S.J. and Thornton J.M. (1993)) using a probe radius of 1.40 Å. Poisson Boltzman ESP was calculated using APBS and PDB2PQC online web server ( http://www.poissonboltzmann.org).

### Paramagnetic relaxation enhancement

Aspartate 31 was converted to Cys in both PEP-19 and PEP(P37G) for site-directed spin labelling with 3-(2-iodoacetamido)-2,2,5,5-tetramethyl-1-pyrrolidinyloxy (PROXYL; Sigma). A ten-fold molar excess of PROXYL dissolved in ethanol was added to Cys derivatives in 40 mM Tris (pH 8), 2 mM EDTA and 6 M urea, and incubated at room temperature in the dark for 4 h. Unreacted PROXYL was then quenched with 10 mM 2-mercaptoethanol and removed by desalting using a Bio-Rad P2 column. Chemical shifts of amide resonances in ^15^N-labelled apo C-CaM bound to spin-labelled PEP-19 (PEP-19SL) or PEP(P37G) (PEP(P37G)SL) are almost identical to those observed when bound to PEP-19 or PEP(P37G), which indicates that the introduction of the PROXYL group does not greatly perturb structure. HSQC spectra were collected at 298 K in the absence and presence of 5 molar equivalents of sodium ascorbate to reduce PROXYL to its diamagnetic form. After linear prediction and zero filling, the spectra were fitted using Lorentzian function in each dimension. The PRE effect was measured as a normalized ratio (*I*_ox_*/I*_red_) of signal intensities in the oxidized (paramagnetic) and reduced (diamagnetic) state.

### Data availability

All relevant data are available from the authors. The assigned chemical shift values, constraints and the atomic coordinates of the final ensemble of 20 structures have been deposited in the Biological Magnetic Resonance Data Bank under accession code 25796 and Protein Data Bank under accession code 2N77.

## Additional information

**How to cite this article:** Wang, X. & Putkey, J. A. PEP-19 modulates calcium binding to calmodulin by electrostatic steering. *Nat. Commun.* **7,** 13583 doi: 10.1038/ncomms13583 (2016).

**Publisher's note**: Springer Nature remains neutral with regard to jurisdictional claims in published maps and institutional affiliations.

## Supplementary Material

Supplementary Information Supplementary Figures 1-8 and Supplementary Table 1

## Figures and Tables

**Table 1:** Experimental restraints and structural statistics for an ensemble of 20 NMR structures for the PEP-19:apo C-CaM complex.

*NMR distance, RDC and dihedral constraints*	
Distance restraints	
Total NOEs	1,926
C-CaM	
Intraresidue (*|i−j|=*0)	599
Sequential (|*i−j*|=1)	280
Medium range (1<|*i−j*|<5)	213
Long range (|*i−j*|≥5)	174
PEP-19 (Phe30–Ser62)	
Intraresidue (*|i−j|=*0)	212
Sequential (|*i−j*|=1)	120
Medium range (1*<*|*i−j*|*<*5)	113
Long range (*|i−j*|≥5)	1
Intermolecular	198
Ambiguous	16
Total predicted hydrogen bonds	98
C-CaM	62
PEP-19 (Phe30–Ser62)	36
Total RDC restraints (H–N)	60
C-CaM	60
PEP-19	0
Total dihedral angle restraints	162
C-CaM	112
PEP-19 (Phe30–Ser62)	50
	
*Structural statistics*	
Violations	
Distance constraints (Å)	0.029±0.002
Dihedral angle constraints (°)	0.34±0.08
RDC constraints (Hz)	0.096±0.013
Max. distance constraint violation (Å)	<0.5
Max. dihedral constraint violation (°)	<5.0
Deviations from idealized geometry	
Bond lengths (Å)	0.002±0.000
Bond angles (°)	0.496±0.005
Impropers (°)	0.429±0.016
Average pairwise RMSD (Å)	
C-CaM (Glu81–Ala128 and Gln135–Thr146)	
Backbone	0.40±0.08
All heavy atoms	1.32±0.10
PEP-19 (Phe30–Lys57)	
Backbone	0.53±0.12
All heavy atoms	1.53±0.15

RDC, residual dipolar couplings; RMSD, root-mean-square deviation.
