# Supplementary material for: PEP-19 modulates calcium binding to calmodulin by electrostatic steering
Source: Nat Commun. 2016 Nov 23;7:13583. doi: 10.1038/ncomms13583 (PMC5122967; doi:10.1038/ncomms13583)
Supplement: Supplementary Information — Supplementary Figures 1-8 and Supplementary Table 1 [file ncomms13583-s1.pdf]

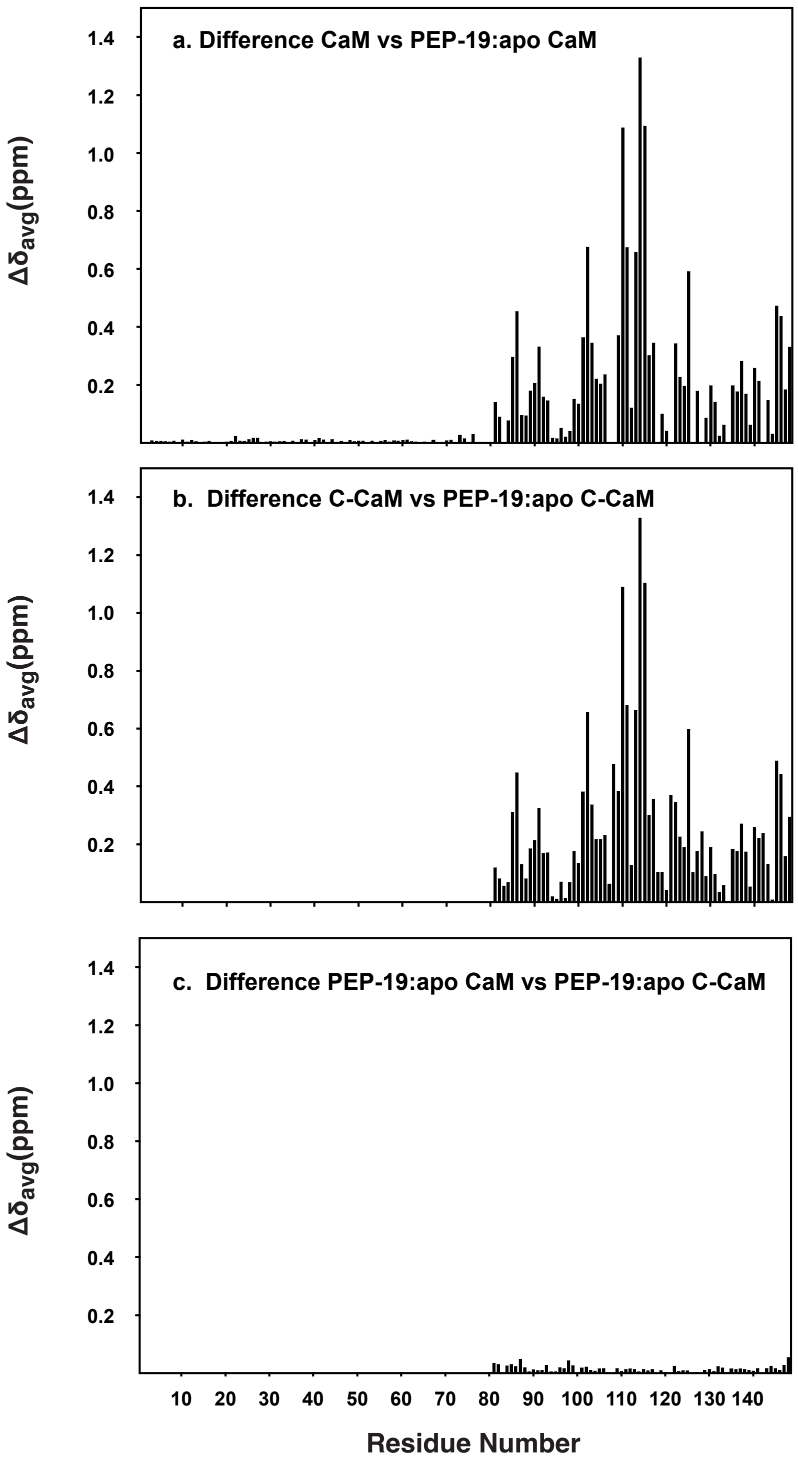

Supplementary Figure 1: PEP-19 binds to the C-domain of CaM to form a 1:1 complex with intact apo CaM or apo C-CaM. ^{1}H, ^{15}N HSQC spectra of ^{15}N-labeled intact apo CaM or apo C-CaM (0.25 mM) were collected at increasing concentrations of PEP-19 up to a 3-fold molar excess. Chemical shift changes were maximal at a 1:1 molar ratio of PEP-19 to CaM or C-CaM. Panel a shows chemical shift perturbations caused by addition of a 2:1 molar ratio of PEP-19 to intact apo CaM. The extremely small change seen for residues 1 to 80, with an average of only 0.007 ppm, demonstrates selective binding of PEP-19 to the C-domain of apo CaM. Panel b shows chemical shift perturbations caused by addition of a 2:1 molar ratio of PEP-19 to apo C-CaM. Similar patterns of chemical shifts are seen for residues 80 to 148 in Panels a and b, which indicates that PEP-19 induces very similar conformational changes in the C-domain of intact apo CaM and apo C-CaM. This is further emphasized in Panel c, which shows backbone amide chemical shift differences between PEP-19: apo CaM versus PEP-19: apo C-CaM complexes. Amide chemical shift changes were calculated using equation (1).

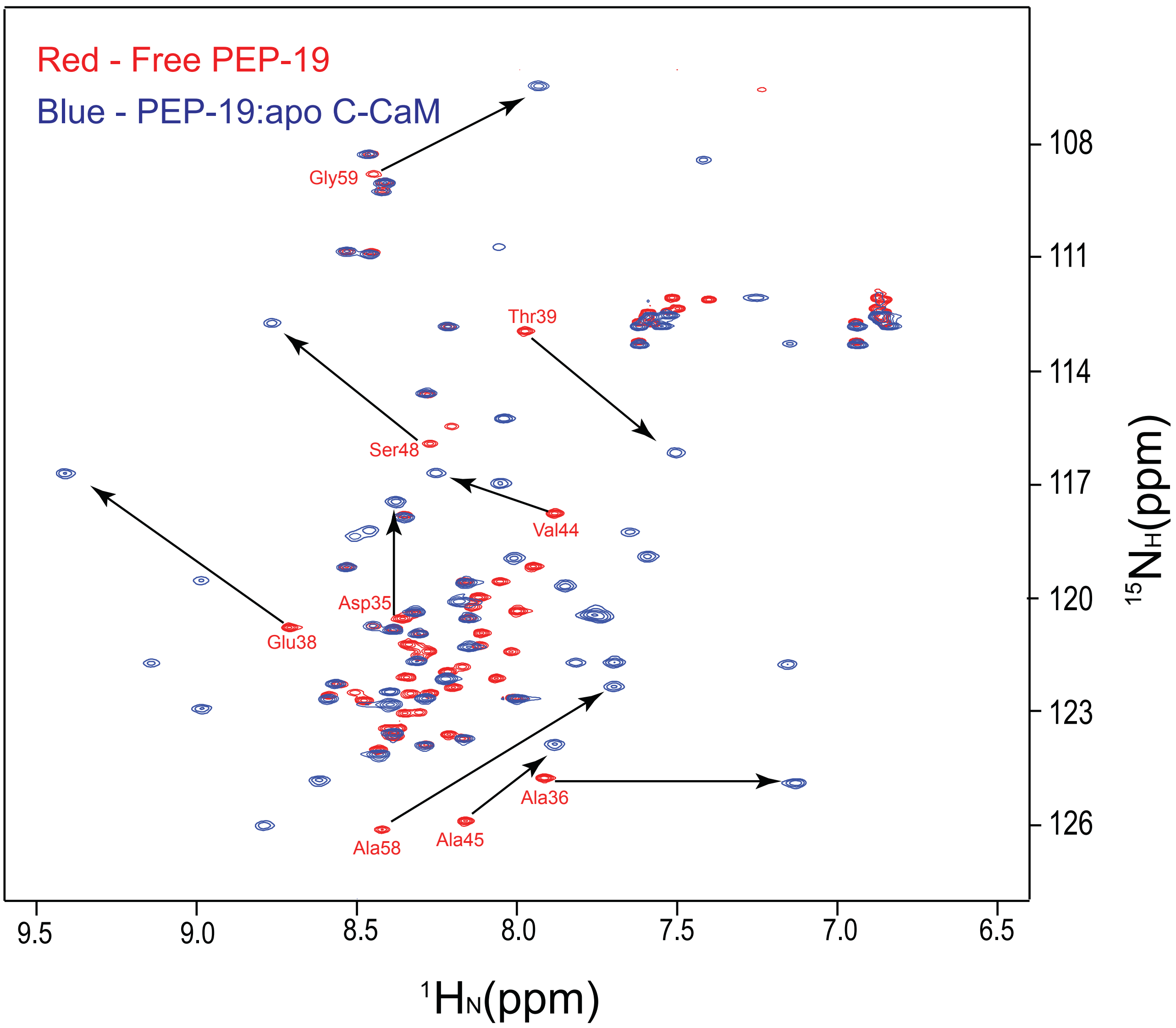

Supplementary Figure 2: Effect of association with apo C-CaM on the structure of PEP-19. ^{1}H, ^{15}N HSQC spectra of ^{15}N PEP-19 collected in the absence (red) and presence (blue) of apo C-CaM are overlaid. The poorly dispersed amide proton resonances and sharp line widths indicate that free PEP-19 is an intrinsically disordered protein. Association with apo C-CaM causes amide ^{1}H resonances from Phe30 to Ala58 in PEP-19 to significantly shift downfield and upfield. The arrows highlight the amide chemical shift changes for selected residues when ^{15}N PEP-19 binds to apo C-CaM.

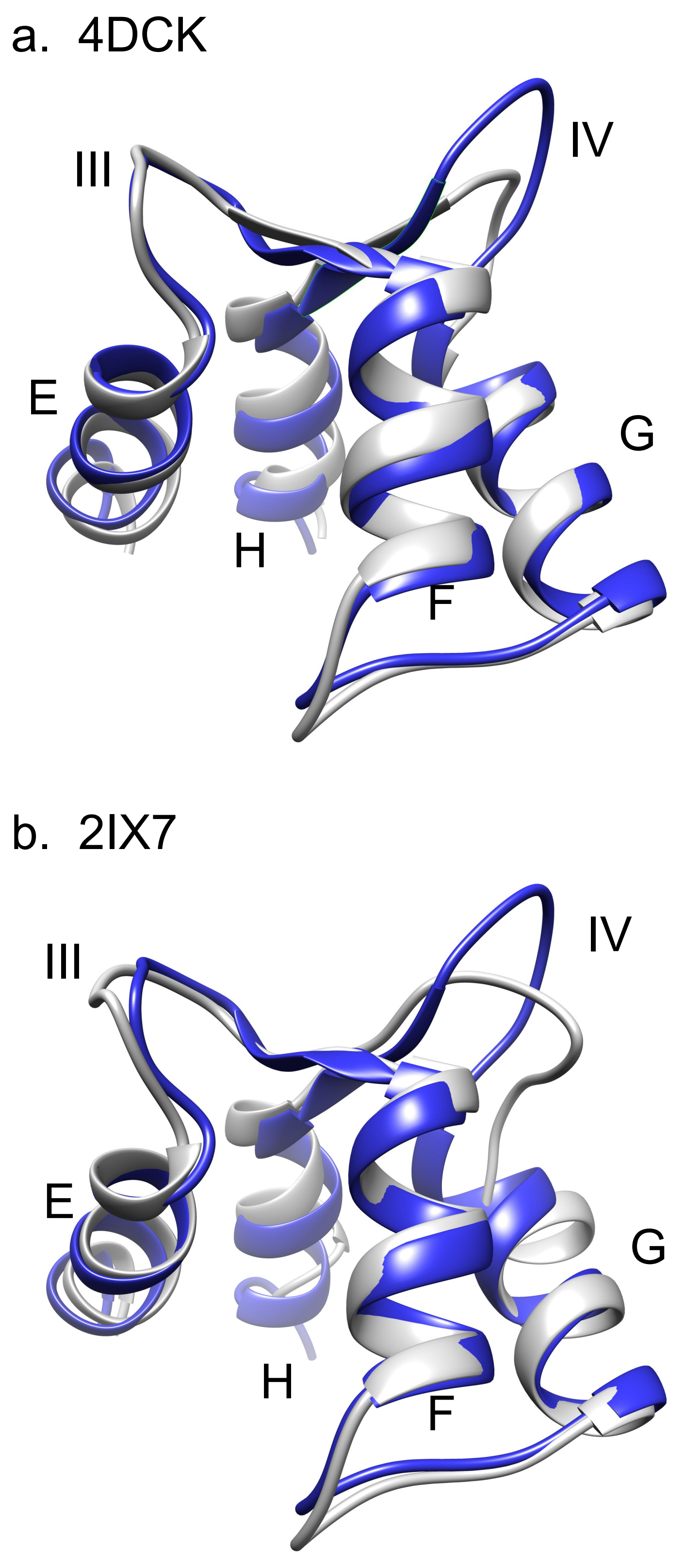

Supplementary Figure 3: Apo C-CaM bound to PEP-19 has a semi-open conformation. Pairwise structural alignments were obtained using the Dali server (http://www.ebi.ac.uk/dali/). *Panel a* and *Panel b* show superimpositions of apo C-CaM from the C-CaM:PEP-19 complex (blue) with the crystal structure of the apo C-domain of CaM (gray) bound to the cytoplasmic domain of the voltage-gated sodium channel $Na_V1.5$ (4DCK; RMSD = 2.2 Å; Z score = 9.5), or the IQ region of myosin V (PDB 2IX7; RMSD = 3.0 Å; Z score = 9.3). Letters E to H indicate helical segments, while III and IV indicate Ca^{2+} binding loops. Superimpositions were generated using Chimera MatchMaker.

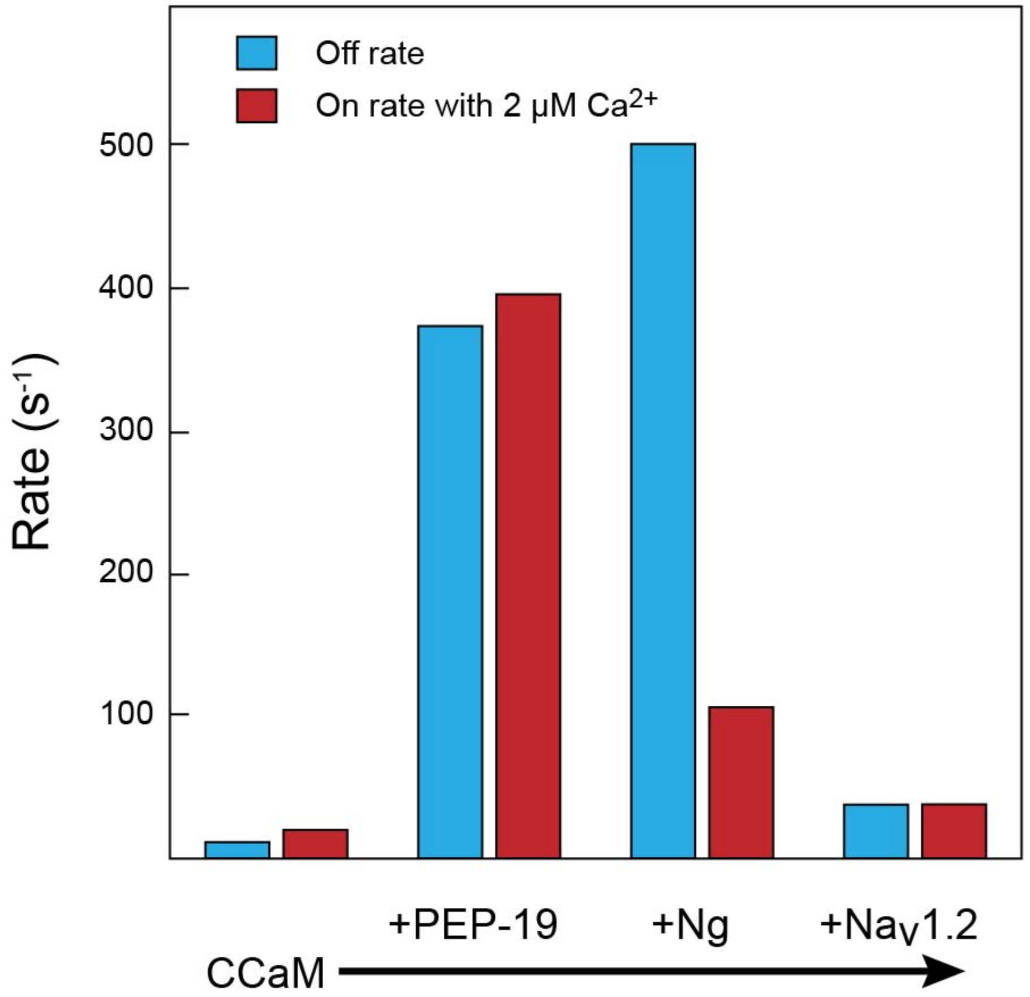

Supplementary Figure 4: The semi-open conformation of C-CaM does not greatly affect the binding of Ca^{2+}. Calcium on- and off rates were measured for free C-CaM, and with bound to intact PEP-19, intact Ng, or the IQ peptide from the voltage-gated sodium channel Na_V1.5 (aa 1901 to 1927). Off-rates (blue bars) were measured using Ca^{2+} sensitive dye Quin-2, and on-rates (red bars) were measured in the presence of 2.0 μM Ca^{2+} using tyrosine fluorescence. Previous structural study showed that Na_V1.5 IQ peptide, which doesn't include the acidic sequence to the N terminus of IQ helix, selectively binds to C-domain of CaM and causes the C-domain of CaM to adopt semi-open conformation [22]. Little effects of Na_V1.5 IQ peptide on Ca^{2+} on- and off rates from C-CaM suggest that the semi-open conformation of apo C-CaM due to IQ peptide binding might not play a role on the significant increased Ca^{2+} binding on-rate to C domain of CaM.

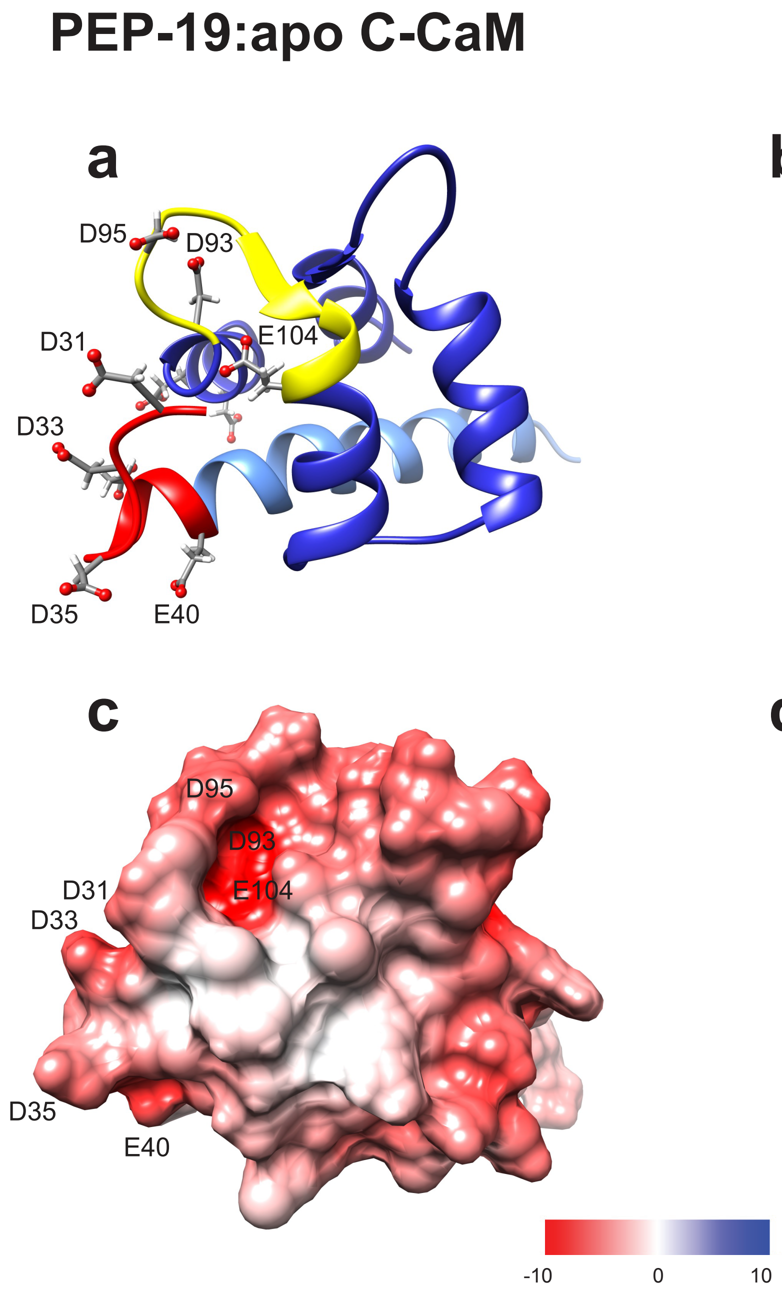

Supplementary Figure 5: The acidic sequence of PEP-19 increases the negative electrostatic surface potential near Asp93 and Glu104 in apo C-CaM. *Panels a and b* show ribbon diagrams for the PEP-19:apo C-CaM complex and free C-CaM, respectively. Dark blue is C-CaM, yellow is Ca^{2+} binding loop III, red and light blue are the acidic sequence and core IQ motif in PEP-19, respectively. *Panels c and d* show solvent excluded surfaces that are colored based on electrostatic surface potential.

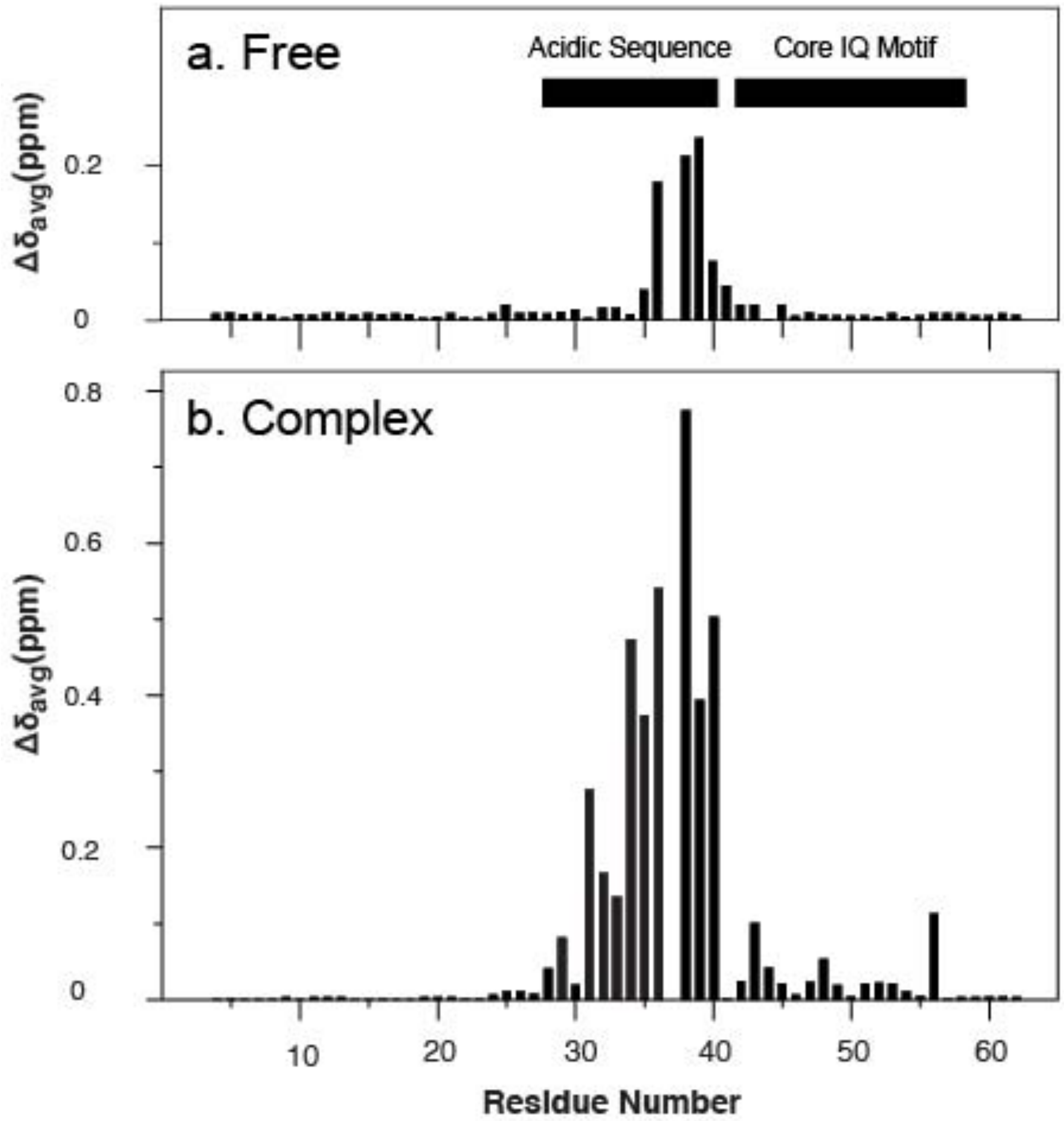

Supplementary Figure 6: Mutation of Pro37 to Gly in PEP-19 causes backbone amide chemical shift perturbations in the acidic sequence when PEP-19 is bound to apo C-CaM. Chemical shift perturbations due to mutation of Pro37 to Gly are shown for free PEP-19 (*Panel a*) and when PEP-19 is bound to apo C-CaM (*Panel b*). Bars indicated the weighted average chemical shift perturbations of amide ^{1}H and ^{15}N calculated using equation (1).

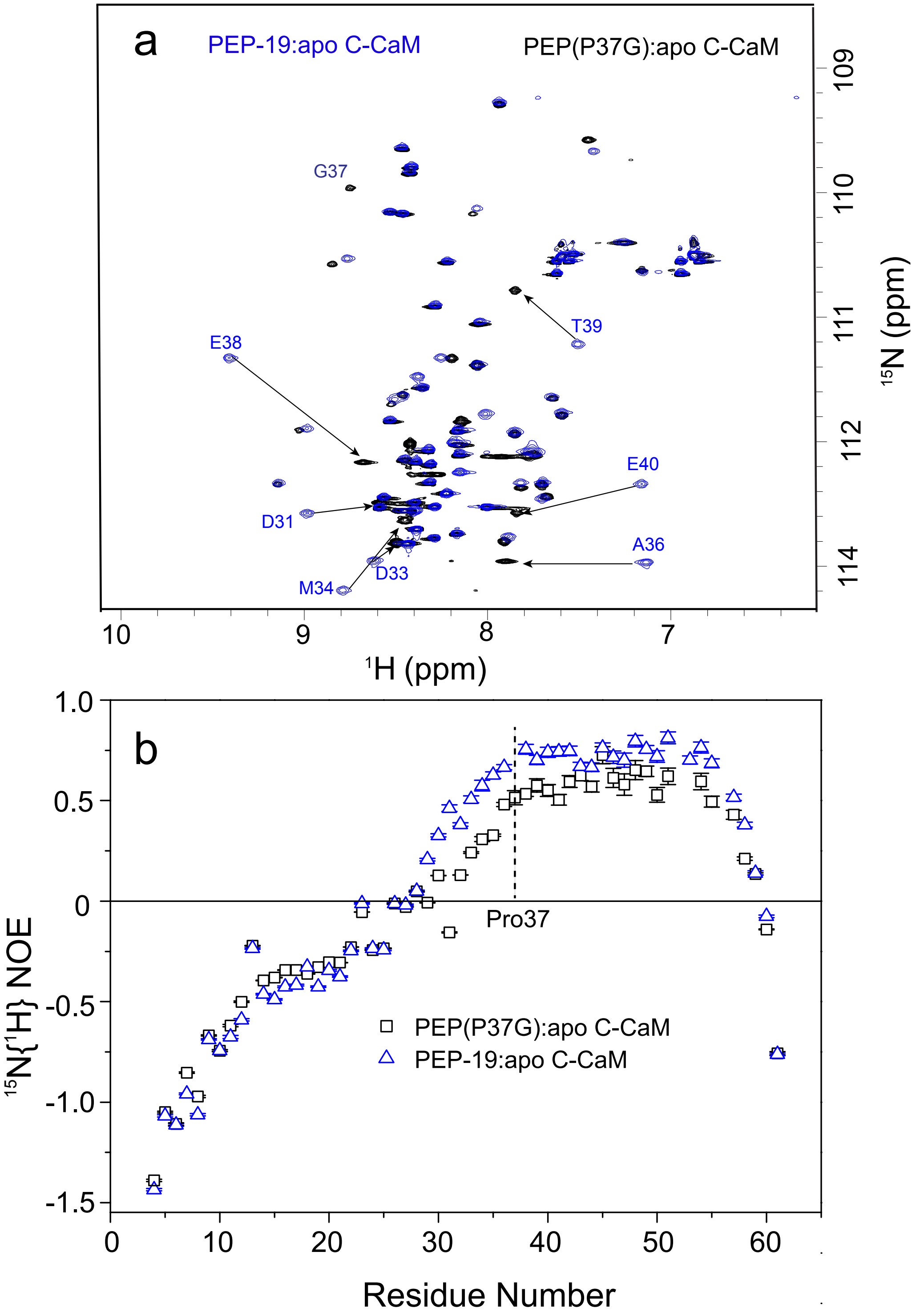

Supplementary Figure 7: Effect of mutation of Pro37 to Gly on ^{1}H, ^{15}N HSQC and heteronuclear ^{15}N{^{1}H} NOEs of PEP-19 bound to apo C-CaM. *Panel a* compares ^{1}H, ^{15}N HSQC spectra of ^{15}N PEP(P37G) (black) and ^{15}N PEP-19 (blue) bound to apo C-CaM. The arrows highlight the amide chemical shift changes from Asp31 to Glu40 in PEP-19 due to the mutation. A significant decrease of dispersion in the ^{1}H dimension indicates that the acidic sequence is less ordered due to the mutation. *Panel b* shows steady-state ^{15}N{^{1}H} NOEs of ^{15}N PEP(P37G) with unlabeled apo C-CaM (squares) and ^{15}N PEP-19 with unlabeled apo C-CaM (triangles). Residues in the unstructured region (aa 4-29) of PEP-19 and PEP(P37G) have very similar ^{15}N{^{1}H} NOE values, with an average difference of only -0.04. The largest difference is seen for residues 28 to 38 in the acid sequence with an average difference of -0.28. The helical IQ sequence (aa 38-62) has an average difference of -0.14. This indicates increased flexibility on ps-ns timescale in the acidic sequence due to the mutation.

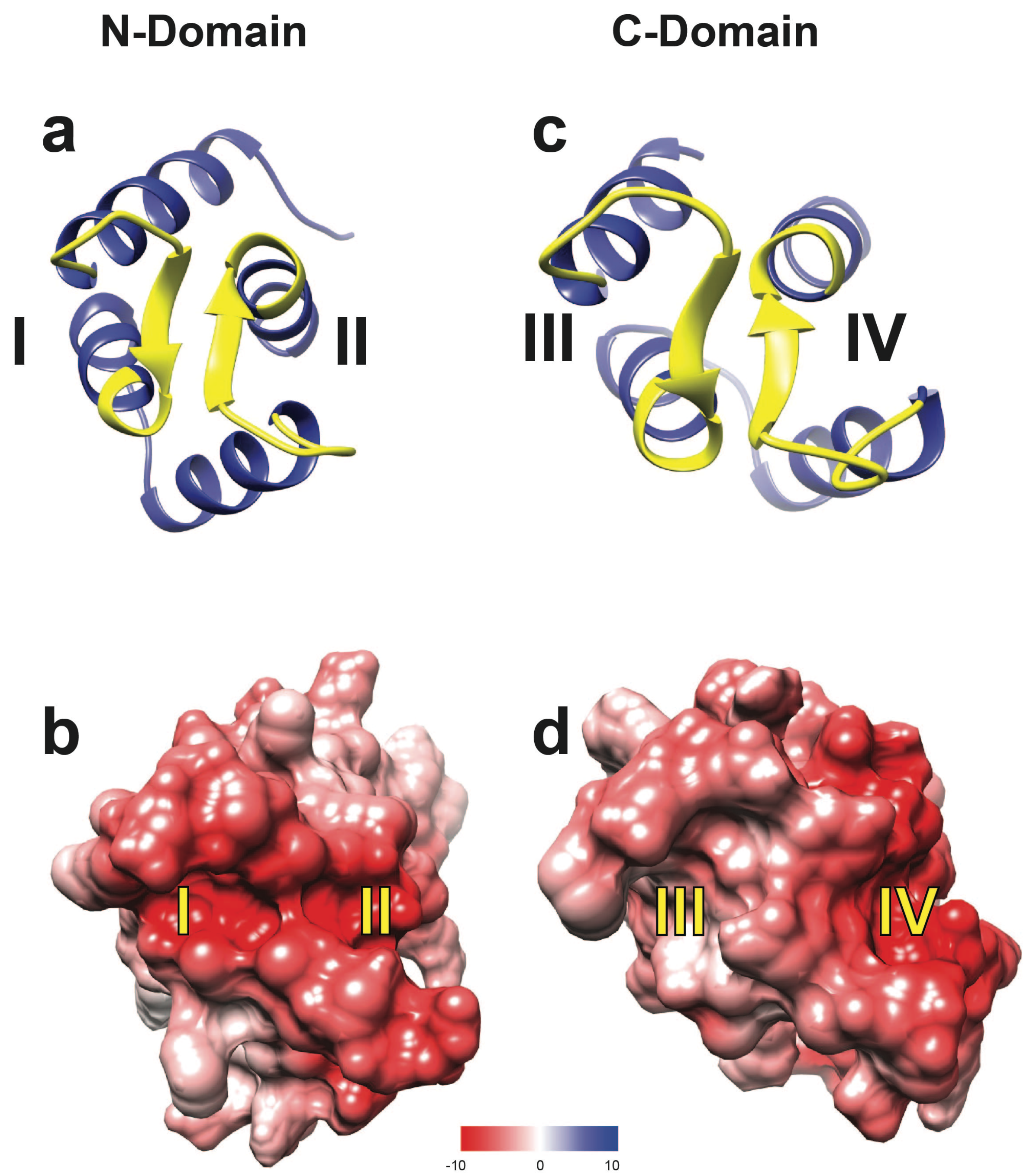

Supplementary Figure 8: Surfaces near Ca^{2+} binding sites I, II, III and IV of free apo CaM have negative electrostatic surface potential. *Panels a and c* show ribbon diagrams of the N- and C-domains of CaM, respectively, with the Ca^{2+} binding loops I, II. III and IV highlighted in yellow. *Panels b and d* show solvent excluded surfaces. Note the extensive area of negative ESP that bridges loops I and II in the N-domain. Loop IV in the C-domain is also surrounded by negative ESP. Loop III is surrounded by substantially more neutral surfaces.

Supplementary Table 1
Interhelical Angles for C-Domain Ca^{2+} Binding Sites in the Ca^{2+}-Bound, Apo and Semi-Open Conformations.

				Helices E/F Site III	Helices G/H Site IV
Ca^{2+}	Open	C-CaM	1J7P	103 ± 2	98 ± 2
Apo	Closed	C-CaM	1F71	133 ± 1	120 ± 1
Apo	Semi-Open	C-CaM/Nav1.5	2L53	102 ± 4	115 ± 6
Apo	Semi-open	C-CaM/PEP-19		101 ± 2	108 ± 4

Helical crossing angles were determined using Chimera, and the above helical angles were calculated from 180-(crossing angle). Values represent the average +/- SD of angles determined for the top 3 to 5 structures in the NMR ensembles.
